# Utilization and associated factors of emergency contraception among women seeking abortion services in health institutions of Dessie town, North East Ethiopia, 2018

**DOI:** 10.1186/s13104-019-4707-0

**Published:** 2019-10-22

**Authors:** Asres Eshetie Feleke, Tewdros Seyoum Nigussie, Tibeb Zena Debele

**Affiliations:** 1 Department of Midwifery, School of Nursing and Midwifery, Wollo University, P.O. Box 1145, Dessie, Ethiopia; 2 Department of Midwifery, College of Medicine and Health Science, University of Gondar, P.O. Box 196, Gondar, Ethiopia

**Keywords:** Emergency contraception, Seeking abortion, North East Ethiopia

## Abstract

**Objective:**

The aim of the study was to assess utilization and associated factors of emergency contraception among women seeking abortion services in Health Institutions of Dessie town, North East Ethiopia, 2018.

**Results:**

Among the interviewed 784 women 400 (51%) utilized emergency contraception. Women’s age of 20–24 years [AOR: 2.10, (95% CI 1.200,3.671)], urban residence [AOR: 2.02 (95% CI 1.273, 3.218], being single [AOR: 1.71, (95% CI 1.176, 2.485)], being knowledgeable on emergency contraception [AOR: 5.66, (95% CI 3.976,8.070)], and having favorable attitude towards emergency contraception [AOR: 2.75 (95% CI 1.946, 3.881)] were significantly associated factors with the utilization of emergency contraceptives. Emphasis has to give on health education on emergency contraceptives focusing on those women who are from the rural area, older and married women.

## Introduction

Emergency contraception refers to birth control modalities that will be used after unprotected sexual intercourse within defined time limits to prevent unwanted pregnancies [1]. Emergency contraceptive is indicated after unprotected sexual intercourse, following sexual abuse, misuse or non-use of contraception [2].

Worldwide, approximately 40 percent of the pregnancies (85 million) were unintended [3]. Women with unintended pregnancy may face the dilemma between terminating the pregnancy or allowing unwanted birth [4].

Every year, In developing countries, at least 22,000 women dies from abortion-related complications [8]. An estimated 620,300 induced abortions were performed in Ethiopia in 2014 [5]. Even if performed safely, abortion might be painful and may have psychological and physical stress for the women [6, 7].

There are two main emergency contraceptives (EC) modalities; these are the Oral emergency contraceptives and intrauterine devices. The oral EC have various degrees of protection against pregnancy according to the time that they were taken. If it is taken within 72 h of unprotected sexual intercourse, they reduce the risks of pregnancy by 95% [8, 9]. Post-coital insertion Cu-IUD within 5–7 days after unprotected sexual intercourse in addition to being an effective method of emergency contraception it can also serve as a safe and effective method of long-term contraception [10].

If emergency contraception are easily available and distributed along with appropriate advocacy activities, millions of unwanted pregnancies and abortions can be averted [11]. Furthermore, the use of EC reduces the cost of expenditures on medical care by preventing unintended pregnancies [8].

In Ethiopia, even though EC pills are distributed free of charge through the support of the non-governmental organization and there are many over the counter preparations for sell, still there is a law utilization of emergency contraceptive pills. Therefore, the aim of this study was to assess utilization and associated factors of emergency contraception among women seeking abortion care services in the health institutions of Dessie town.

## Main text

### Methods

#### Study design, period and setting

An institutional-based cross-sectional study was conducted from August 2, 2018, to January 30, 2019 in Dessie town health institutions. Dessie town is located at 401 km from the capital city of Ethiopia, Addis Ababa. The town has five Hospitals (one public referral, one public general and three private), eight health centers and twenty-seven private clinics. Currently, there are 10 health institution fully providing safe abortion care services for the community. In those health institutions, 6 months prior to the data collection period, there were 2830 safe abortion cases.

#### Sample size and sampling procedure

To estimate a sample size of 784 participants were calculated with single population proportion formula with the following assumptions. The magnitude of EC utilization 9.7% from a previous study done in Diredawa [12], 95% confidence interval, 3% margin of error, a design effect of 2 and non response rate of 10%. Initially, 10 health facilities that provide abortion service were stratified into 5 governmental and 5 private institutions. Then after, out of the 5 institutions, 3 were selected from each group by simple random sampling technique. After proportionally allocating the total numbers of the participants, a systematic random sampling method was employed to select the actual study participants.

#### Operational definitions

Utilization of EC: if a woman has any history of EC usage [13, 14].

Knowledgeable: Respondents who scored above the mean value of the knowledge assessment questions [15].

Favorable attitude: Respondents who scored above the mean value of attitude assessment questions [15, 16].

#### Data collection instrument and process

Data was collected by face to face interview using a semi-structured and pre-tested questionnaire which is prepared first in English and translated to Amharic and then translated back to English to assure consistency. Ten BSc midwifery holders were involved in the data collection while two MSc holders supervised the data collection process.

#### Data analysis

Data were entered with epi-info version7 and analyzed using SPSS version 20 software. Bivariate and multivariable logistic regression analyses were conducted to identify predictors for emergency contraceptive utilization. Variables found to be significant on Bivariate analysis (p < 0.2) were included in the multivariable model. Adjusted odds ratios and 95% confidence intervals were generated. p-value < 0.05 were considered statistically significant.

### Results

#### Socio-demographic characteristics

A total of 784 women were involved in the study making a response rate of 100%. The mean age of the respondents was 24.28 years with SD of ± 4.74. The majority of 621 (79.2%) were urban residents, and 420 (53.6%) were single (Table 1).

**Table 1:** Socio demographic characteristics of women seeking abortion services in health institutions of Dessie town, 2018 (n = 784)

Variables	Frequency	Percent
Age (years)		
13–19	130	16.6
20–24	285	36.4
25–29	259	33
≥ 30	110	14
Residence		
Urban	621	79.2
Rural	163	20.8
Marital status		
Single	501	63.9
Married	283	36.1
Religion		
Muslim	420	53.6
Orthodox	344	43.9
Others	20	2.5
Ethnicity		
Amhara	719	91.7
Oromo	29	3.7
Others	36	4.6
Educational status		
No primary education	55	7
Primary education	180	23
Secondary education	317	40.4
Tertiary education	232	29.6
Father’s educational status		
No primary education	480	61.2
Primary education	105	13.4
Secondary education	110	14
Tertiary education	89	11.4
Mother’s educational status		
No primary education	614	78.3
Primary education	83	10.6
Secondary education	54	6.9
Tertiary education	33	4.2
Occupation		
Housewife	134	17
Merchant	110	14
Government employed	141	18
Student	285	36.4
Servant	86	11
Commercial sex worker	28	3.6
Living arrangement		
With family	275	35.1
With spouse	209	26.6
With peers in a rented house	60	7.7
Alone	165	21
Others	75	9.6
Income		
< 500 ETB	317	40.4
501–1000 ETB	162	20.7
1001–1500 ETB	72	9.2
1501–2000 ETB	92	11.7
Above 2000 ETB	141	18

Others for religion represent Catholic, Protest

Others for ethnicity Tigray, South nations nationalists

Others for living arrangement, with a relative, in campus

#### Sexual and reproductive characteristics of respondents

Four hundred three (51.4%) of respondents had started first sexual intercourse less than the age of 18. Among the respondents, 342 (43.6%) have one or more children. Six hundred four (77%) were first time arrival for termination of pregnancy, 179 (22.8) had a prior history of induced abortion, among those who had a history of induced abortion, 135 (75.4%) had one episode, and 28 (15.6%) had more than three induced abortions.

#### Distance from home to the nearby health institution (in terms of time elapse)

The respondent’s distance from home to the nearby health institution ranges from 1 min to 720 min. Majority of the respondents 638 (81.4%) stated that stated they could reach in the nearby health institution within 30 min.

#### Utilization of regular family planning methods

Five hundred forty-one (69%) had ever used regular contraceptive methods. Among those who had ever used regular contraceptive method 397 (73.4%) used an injectables contraceptive.

#### Knowledge and attitude about emergency contraceptive

The overall summary index for knowledge and attitude of the respondents about EC disclosed that 434 (55.4%) were knowledgeable and 376 (48%) had a favorable attitude towards EC.

#### Utilization of emergency contraception

In this study 400 (51%) of respondents had ever used EC; were all of them used EC pills. The main source of information for those who ever used EC were friends 243 (60.8%), sexual partner 84 (21%), media 50 (12.5%), health professionals 6 (1.5%), web pages 7 (1.7%) and 10 (2.5%) other sources,

Respondents who had never used EC mentioned main factors for non-utilization and they are; lack of information 255 (66.5%), time inconvenience 14 (3.7%), lack of willingness 19 (4.9%), drugs unavailability 5 (1.3%), privacy issue 6 (1.5%).

#### Factors associated with the utilization of emergency contraception

On the bivariate analysis factors that significantly associated with EC use were: women’s age of 20–24 years, urban residence, being orthodox by religion, being single by marital status, father’s educational status more than primary and above, having one children and above, knowledgeable on EC having favorable attitude towards EC From In multivariate binary logistic regression analysis; age of respondents 20–24 years [AOR = 2.10, 95% CI 1.200–3.671], urban residence [AOR = 2.02, 95% CI 1.273–3.218], being single by marital [AOR = 1.71, 95% CI 1.176–2.485], knowledgeable on EC [AOR = 5.66, 95% CI 3.976, 8.070], having favorable attitude towards EC [AOR = 2.75, 95% CI 1.946–3.881] were found to be significantly associated with EC (Table 2).

**Table 2:** Bivariate and multivariate logistic regression analysis of factors associated with EC use among women seeking abortion service in Health Institutions of Dessie town, North East Ethiopia, 2018 (n = 784)

Utilization	Utilization		COR (95% CI)	AOR (95% CI)
	Yes (n = 400)	No (n = 384)		
Age (years)				
13–19	55	75	1.28 (0.76, 2.16)	
20–24	171	114	2.62 (1.67, 4.14)	2.10 (1.200, 3.671)
25–29	134	125	1.88 (1.19, 2.97)	
≥ 30	40	70	1	
Residence				
Rural	47	116	1	
Urban	353	268	3.25 (2.24, 4.73)	2.02 (1.273, 3.218)
Marital status				
Married	129	154	1	
Single	271	230	1.40 (1.05, 1.89)	1.71 (1.176, 2.485)
Number of children				
None	240	202	1	
One and above	160	182	0.74 (0.56, 0.98)	
Religion				
Muslim	193	227	1	
Orthodox	193	151	1.50 (1.13, 2.00)	
Other	14	6	2.74 (1.03, 7.23)	
Ethnicity				
Amhara	357	362	0.56 (0.28, 1.12)	
Oromo	20	9	1.26 (0.44, 3.55)	
Other	23	13	1	
Father’s educational status				
No primary education	204	276	1	
Primary education	64	41	2.11 (1.37, 3.25)	
Secondary education	71	39	2.46 (1.60, 13.79)	
Tertiary education	61	28	2.95 (1.82, 4.78)	
Knowledge				
Not knowledgeable	82	268	1	
Knowledgeable	318	116	8.96 (6.47, 12.41	5.66 (3.976, 8.070)**
Attitude				
Unfavorable attitude	140	268	1	
Favorable attitude	260	116	4.29 (3.18, 5.89)	2.75 (1.946, 3.881)**

** p-value < 0.05

### Discussion

This study finds out that 400 (51%) of women who came for abortion service had ever used EC (95% CI 47.9, 54.6). This is in agreement with the study that was done among abortion care seekers in China (48.8%) [17].

This finding is lower than the studies that were done in North India (70%) in Durban South Africa (62.1%) and among women of the reproductive age group in India [18–20]. Possible reasons for this difference might be due to the differences in the study area, study population, and knowledge about EC. In the North Indian study, the participants were recently married and data were collected from a single government health institution. Additionally, their knowledge score was also higher than the current study. In the Durban study, the main source of information about EC were health care professional’s contrary to the current study where the majority of the respondents obtained information about EC from their friends who might not share correct information that intern leads to low utilization.

This finding is higher than studies that were done among abortion seekers in Jimma specialized hospital were none of them ever used EC, Dire-dawa (9.7%) and India (1.155%) had ever used EC [12, 21, 22]. this difference might be due to the difference in socio-demographic, cultural or developmental differences of the study with India. In the Jimma and Diredawa study, the knowledge and attitude of the participants were lower than the current study, which may reduce utilization of EC.

Women’s age was a significant factor to EC use. Women in their age 20–24 years were 2.10 times more likely utilized EC as compared with women aged ≥ 30 years. This is in agreement with the studies conducted among abortion seekers in Dire-dawa [12], and South Africa [23]. Possible reasons for this might be women in this age group might not use regular contraceptive methods consistently due to cultural influence which might lead them to use EC. In addition, this group of women is mostly college students that might have the opportunity to get information about EC after unprotected sexual intercourses from their peer which intern increase the utilization.

Residence was an important determining factor for EC use. Those women who were living in an urban area were 2.02 times more likely utilized EC as compared with those who were living in the rural area. This is consistent with the study that was done in Arbaminch [24]. The possible reasons might be women living in urban areas could be more exposed to media, which might create awareness about family planning methods including EC. Another explanation could be the fact that EC is more accessible in the urban areas than their rural counterparts. On the contrary, a shortage of media coverage in the rural area could have a negative influence utilization of EC for women living in the rural area.

Marital status was significantly associated with EC use. Women who were single were 1.71 times more likely to utilized EC as compared with those who were married. This is in agreement with the study that was done in Dire-dawa [22]. The possible explanation could be single women might not use regular contraceptive methods consistently which increases the utilization. In addition, single women could have unplanned sexual intercourse not feasible to use regular contraceptive methods which intern leads to more utilization of EC.

Women who were knowledgeable about EC were 5.66 times more likely to utilize. This is coherent with studies that were done among abortion seekers in Dire-dawa [22], South Africa [24]. This might be explained by knowing effectiveness, where they can get when they can use EC may help to use EC.

Attitude towards EC was significantly associated with the use of EC. Participates who had a favorable attitude towards EC were 2.75 times more likely to utilized EC. This is consistent with studies done in Debre-Markos higher institutions [25]. The above reports might be explained with women who have a favorable attitude towards EC might want to know more about EC and to use it.

## Limitations of the study

Questions that had sensitive nature like age of first sexual intercourse, history of previous induced abortion might create social desirability bias.

## Data Availability

The authors declare that the data regarding this manuscript can be accessed as per the request of any interested body and can be submitted for publication in Spring Nature as supplementary materials.

## Abbreviations

AOR: adjusted odds ratio
CI: confidence interval
COR: crude odds ratio
Cu-IUD: copper bearing intrauterine device
EC: emergency contraceptive
